# Corticosteroid use in oral surgery: A review of dosing protocols and clinical outcomes

**DOI:** 10.6026/973206300220021

**Published:** 2026-01-31

**Authors:** Aditya Narayan Shukla, Pramod D.S Raghavendra, Sonali Perti, Sapna Pandey, Vishwannath Hiremath, Syed Sajid Basha

**Affiliations:** 1 Department of Oral & Maxillofacial Surgery, Babu Banarasi Das College of Dental Sciences, Lucknow, Uttar Pradesh, India; 2 Department of Oral & Maxillofacial Surgery, Riyadh Health cluster Ministry of Health, Saudi Arabia; 3 Department of Prosthodontics, Kalinga Institute of Dental Sciences, KIIT University, Patia, Bhubaneswar, Odisha, India; 4 Department of Oral & Maxillofacial Surgery, Chandra Dental College and Hospital, Uttar Pradesh, India; 5 Department of Oral and Maxillofacial Surgery, A Unit of Hiremath Hospitals Pvt Ltd, Vijayanagar, Bangalore, India; 6 Department of Oral and Maxillofacial Surgery, Maharana Pratap College of Dentistry and Research Centre, Gwalior, India

**Keywords:** Corticosteroids, dexamethasone, oral surgery, postoperative complications, pain management, edema, trismus, narrative review

## Abstract

Corticosteroids have become integral components of perioperative management in oral surgery, offering significant potential for reducing postoperative complications including pain, swelling, and trismus. Despite widespread clinical adoption, considerable variability exists in prescribing patterns regarding drug selection, dosing protocols, routes of administration, and timing of delivery. Available data shows that dexamethasone, particularly at doses of 4-16 mg, effectively reduces postoperative morbidity when compared to placebo. Thus, preoperative administration appears to offer superior outcomes compared to postoperative dosing, supporting a preemptive anti-inflammatory approach.

## Background:

Postoperative morbidity following oral surgical procedures remains a significant clinical concern, with patients commonly experiencing a triad of complications comprising pain, edema and trismus [1]. These inflammatory sequelae arise from the complex interplay of tissue trauma, surgical manipulation and the subsequent release of inflammatory mediators including prostaglandins, bradykinins and various cytokines [2]. The effective management of these postoperative complications is paramount to ensuring optimal patient recovery, satisfaction and quality of life during the healing period [3]. Corticosteroids represent a class of potent anti-inflammatory agents that exert their therapeutic effects through multiple pharmacological mechanisms. These include inhibition of phospholipase A2 enzyme activity, reduction of capillary permeability, suppression of leukocyte migration and chemotaxis, and downregulation of pro-inflammatory cytokine expression [4]. Such comprehensive anti-inflammatory properties provide a strong theoretical foundation for the application of corticosteroids in managing postoperative inflammation following oral surgical interventions. In contemporary clinical practice, corticosteroid administration has been adopted across a broad spectrum of oral surgical procedures, including third molar extractions, dental implant placement, orthognathic surgery, and various other dentoalveolar operations [5]. Despite the widespread utilization of corticosteroids in oral surgery, substantial heterogeneity exists in prescribing patterns among oral and maxillofacial surgeons worldwide [6]. This variation encompasses multiple dimensions of corticosteroid therapy, including the choice of specific agent (dexamethasone, methylprednisolone, prednisolone, or betamethasone), dose selection (ranging from 4 mg to 125 mg depending on the formulation), route of administration (oral, intramuscular, intravenous, or submucosal), timing relative to surgery (preoperative, intraoperative, or postoperative), and duration of therapy (single dose versus multiple-day regimens) [7]. This lack of standardization reflects both the absence of universally accepted evidence-based practice guidelines and the sometimes contradictory findings reported across individual studies [8].

Previous reviews have examined the efficacy of corticosteroids in specific oral surgical contexts, particularly third molar extraction and have generally supported their use while acknowledging significant heterogeneity in protocols and outcomes [9, 10]. Recent investigations have suggested potential advantages of preoperative administration compared to postoperative dosing, although definitive consensus on this matter remains elusive [11]. Similarly, the optimal dosing strategy continues to be debated, with some researchers advocating for higher doses to maximize anti- inflammatory effects while others emphasize the importance of minimizing doses to reduce potential adverse effects [12]. Safety considerations represent critical clinical determinants influencing corticosteroid prescribing decisions. While short-term corticosteroid use is generally well-tolerated in healthy individuals, potential concerns include hyperglycemia, immunosuppression, delayed wound healing, adrenal axis suppression, and gastrointestinal complications [13]. Establishing evidence-based dose-response relationships and identifying minimal effective dosing strategies are essential for optimizing the balance between therapeutic efficacy and safety profiles [14]. Significant knowledge gaps persist regarding optimal corticosteroid regimens in oral surgery. Comprehensive evaluation of comparative efficacies among different agents, dose-response characteristics, ideal timing and routes of administration, and safety profiles across diverse patient populations remains an ongoing clinical need. Furthermore, the relationship between specific dosing protocols and the magnitude of clinical benefit has not been thoroughly analyzed, limiting the translation of available evidence into practical clinical recommendations [15].

## Pharmacological basis for corticosteroid use in oral surgery:

Understanding the pharmacological properties of corticosteroids is fundamental to appreciating their role in managing postoperative inflammation. Corticosteroids exert their anti-inflammatory effects primarily through genomic mechanisms involving the glucocorticoid receptor, a nuclear receptor that modulates the transcription of numerous genes involved in inflammatory processes [4]. Upon binding to their intracellular receptors, corticosteroids translocate to the nucleus where they suppress the expression of pro- inflammatory genes encoding cytokines, chemokines, adhesion molecules, and inflammatory enzymes. The inhibition of phospholipase A2 represents a particularly important mechanism in the context of surgical inflammation. This enzyme catalyzes the release of arachidonic acid from membrane phospholipids, serving as the rate-limiting step in the production of prostaglandins, leukotrienes, and other eicosanoids that mediate pain, swelling, and inflammation [2]. By suppressing phospholipase A2 activity, corticosteroids effectively block the synthesis of these potent inflammatory mediators at a point upstream of both cyclooxygenase and lipoxygenase pathways.

Additionally, corticosteroids reduce capillary permeability and inhibit the extravasation of fluid and plasma proteins into injured tissues, directly counteracting the formation of inflammatory edema [6]. They also suppress the recruitment, activation, and function of various inflammatory cells including neutrophils, macrophages, and lymphocytes, further attenuating the inflammatory response. These combined effects provide a robust pharmacological rationale for corticosteroid use in minimizing postoperative inflammatory complications. Among the various corticosteroids available for clinical use, dexamethasone and methylprednisolone have emerged as the most commonly employed agents in oral surgery [7]. Dexamethasone offers several practical advantages including high glucocorticoid potency, minimal mineralocorticoid activity, excellent oral bioavailability, and a prolonged biological half-life of 36-72 hours that permits effective single-dose administration. Methylprednisolone, while having a shorter half-life of 18-36 hours, provides comparable anti-inflammatory efficacy and is available in various formulations suitable for parenteral administration.

## Corticosteroid efficacy in reducing postoperative pain:

Postoperative pain represents one of the most immediate concerns for patients undergoing oral surgical procedures and significantly impacts recovery experience and satisfaction. The analgesic effects of corticosteroids, while not direct in the manner of traditional analgesics, derive from their anti-inflammatory properties and their ability to reduce the production of pain-sensitizing mediators at the site of tissue injury [1]. The literature consistently demonstrates that perioperative corticosteroid administration produces meaningful reductions in postoperative pain intensity across various oral surgical procedures. Studies evaluating dexamethasone, the most extensively investigated corticosteroid in this context, have reported significant decreases in pain scores measured using validated instruments such as the Visual Analog Scale (VAS) and Numerical Rating Scale (NRS) [3]. The magnitude of pain reduction appears most pronounced during the first 24-48 hours following surgery, coinciding with the peak of the acute inflammatory response.

Research examining dose-response relationships for pain reduction has yielded important insights. Lower doses of dexamethasone (less than 8 mg) demonstrate modest but statistically significant analgesic benefits, while doses of 8 mg or greater produce more substantial pain relief [8]. However, the incremental benefit of doses exceeding 8 mg appears to plateau, suggesting that this dose may represent an optimal threshold for balancing efficacy and minimizing unnecessary corticosteroid exposure. Studies investigating methylprednisolone at doses ranging from 40-125 mg have reported comparable analgesic efficacy, indicating that the choice between these agents may be guided by practical considerations rather than differential effectiveness [9]. The timing of corticosteroid administration relative to surgery has emerged as a critical determinant of analgesic efficacy. Preoperative administration, typically 30-60 minutes before the surgical incision, consistently demonstrates superior pain control compared to postoperative dosing [11]. This finding supports the concept of preemptive analgesia, wherein the establishment of anti-inflammatory effects prior to tissue injury may prevent the amplification of nociceptive signaling and central sensitization that characterize postoperative pain states. The preventive approach allows corticosteroids to modulate the initial inflammatory cascade rather than attempting to suppress an already established inflammatory response. Beyond reducing pain intensity scores, corticosteroid administration has been associated with decreased consumption of rescue analgesic medications during the postoperative period [10]. This reduction in analgesic requirements provides additional evidence of clinically meaningful pain relief and may offer secondary benefits including reduced exposure to opioid medications and their associated side effects. The convergence of evidence from pain intensity assessments and analgesic consumption patterns strengthens confidence in the genuine analgesic value of perioperative corticosteroid therapy.

## Effects on facial swelling and edema:

Facial swelling following oral surgery represents not only a source of physical discomfort but also a significant concern for patients due to its visible nature and impact on social interactions during the recovery period [11]. The anti-edema effects of corticosteroids derive from their ability to reduce capillary permeability, decrease inflammatory exudate formation, and suppress the recruitment of inflammatory cells that contribute to tissue swelling. Investigations into corticosteroid effects on postoperative edema have employed various measurement techniques ranging from simple linear facial measurements to sophisticated three- dimensional facial scanning technologies. Despite methodological heterogeneity, the literature demonstrates consistent and substantial reductions in facial swelling with corticosteroid administration compared to placebo or no treatment [4]. The magnitude of swelling reduction is clinically meaningful, with studies reporting percentage decreases ranging from 5% to 15% compared to control groups at peak swelling timepoints, typically 48-72 hours postoperatively. Similar to findings for pain outcomes, preoperative corticosteroid administration appears to offer superior anti-edema effects compared to postoperative dosing [5]. This temporal relationship again supports the value of establishing anti-inflammatory conditions prior to surgical trauma. By preventing the initial inflammatory cascade rather than attempting to reverse established inflammation, preoperative corticosteroids may more effectively limit the extent and duration of postoperative edema. Dose-response relationships for anti-edema effects parallel those observed for pain outcomes. Studies employing dexamethasone at doses of 8 mg demonstrate robust reductions in facial swelling, while both lower and higher doses show effectiveness with varying magnitudes of benefit [12]. Methylprednisolone at conventional doses (40-125 mg) produces comparable anti-edema effects, further supporting the interchangeability of these agents based on practical considerations. The clinical significance of reduced facial swelling extends beyond cosmetic concerns. Excessive edema may compromise the surgical site, potentially affecting wound healing and increasing the risk of complications. Furthermore, facial swelling can cause secondary functional impairments including difficulty with oral intake, speech alterations, and interference with daily activities [11]. By attenuating postoperative swelling, corticosteroids contribute to improved overall recovery experience and earlier return to normal function.

## Impact on trismus and functional recovery:

Trismus, defined as restricted mouth opening due to muscle spasm or inflammation affecting the muscles of mastication, represents a common and functionally significant complication of oral surgery, particularly procedures involving the posterior mandible [12]. The limitation in mouth opening impairs essential functions including eating, oral hygiene maintenance, and speech, significantly impacting quality of life during the recovery period. Corticosteroid administration has been shown to produce meaningful improvements in maximum mouth opening during the postoperative period. Studies measuring interincisal distance report increases of several millimeters in patients receiving corticosteroids compared to controls, representing clinically significant functional improvements [10]. These benefits are evident within the first 48-72 hours following surgery and may persist throughout the early recovery period. The mechanism underlying improved mouth opening likely relates to the combined effects of reduced inflammatory swelling affecting the masticatory muscles and decreased pain that otherwise limits voluntary jaw movement. By attenuating inflammation in the surgical field and surrounding tissues, corticosteroids create conditions more favorable for early functional recovery. The reduction in protective muscle guarding secondary to pain relief further facilitates improved mouth opening. The functional benefits of improved mouth opening have practical implications for patient recovery. Adequate jaw mobility is essential for maintaining oral hygiene through tooth brushing and rinsing, preventing secondary complications such as wound infection or dry socket [13]. Similarly, the ability to consume adequate nutrition supports healing and overall recovery. Patients with less trismus report higher satisfaction with their surgical experience and perceive faster recovery.

## Route of administration considerations:

Corticosteroids may be administered via multiple routes in the perioperative setting, including oral, intravenous, intramuscular, and local submucosal injection. Each route offers distinct advantages and limitations that influence clinical decision-making [7]. ]Oral administration represents the simplest and most convenient route, offering ease of use, patient acceptance, and suitability for outpatient settings. Dexamethasone demonstrates excellent oral bioavailability, making this route pharmacokinetically appropriate for achieving therapeutic systemic concentrations. The primary limitation of oral administration relates to the time required for absorption and distribution to achieve peak tissue concentrations, necessitating administration well in advance of surgery for optimal preoperative dosing. Parenteral routes including intravenous and intramuscular injection provide more rapid onset of action and ensure complete drug delivery independent of gastrointestinal absorption [6]. These routes may be preferred in hospital or surgical center settings where intravenous access is established for other purposes. Intravenous administration offers the most rapid achievement of peak plasma concentrations, while intramuscular injection provides reliable absorption with slightly delayed peak levels. Local submucosal injection of corticosteroids adjacent to the surgical site represents an alternative approach that delivers high local drug concentrations directly to the area of tissue trauma [14]. This technique theoretically maximizes local anti-inflammatory effects while minimizing systemic exposure. Studies evaluating submucosal dexamethasone injection have demonstrated efficacy in reducing postoperative morbidity, though whether this approach offers advantages over systemic administration remains incompletely resolved. The available evidence suggests that efficacy is generally comparable across administration routes when appropriate doses are employed, suggesting that route selection may be guided primarily by practical considerations including clinical setting, available resources, and operator preference [7]. However, the importance of preoperative timing appears consistent regardless of administration route, emphasizing the value of establishing anti-inflammatory effects before surgical trauma occurs.

## Safety profile and adverse effects:

The safety of perioperative corticosteroid administration represents a critical consideration in clinical decision-making. Concerns regarding potential adverse effects including delayed wound healing, increased infection risk, hyperglycemia, and adrenal suppression have historically limited corticosteroid adoption among some practitioners [13]. The available evidence from clinical trials consistently demonstrates that short-term, single-dose or brief-course corticosteroid regimens used in oral surgery are well-tolerated with minimal adverse effects in healthy patients [8]. Adverse event rates in corticosteroid-treated patients are generally comparable to those observed in control groups, indicating no significant increase in complications attributable to corticosteroid therapy. Specific concerns regarding wound healing complications appear largely unfounded in the context of short-term perioperative use. Studies have not demonstrated clinically significant delays in surgical wound healing or increased rates of wound complications such as dehiscence or infection with conventional corticosteroid dosing regimens [14]. The anti-inflammatory effects of corticosteroids, rather than impairing healing, may actually support optimal tissue repair by limiting excessive inflammatory responses that can be detrimental to wound healing. The risk of clinically significant hyperglycemia is minimal in non-diabetic patients receiving short-course corticosteroid therapy. In patients with diabetes mellitus, transient elevations in blood glucose may occur, warranting appropriate monitoring and management but not necessarily precluding corticosteroid use when clinical benefits are anticipated [13]. Careful patient selection and perioperative glucose management protocols can minimize this concern in diabetic patients undergoing oral surgery. Adrenal suppression from single-dose or brief corticosteroid courses is not clinically significant and does not require tapering regimens or supplemental steroid coverage [15]. The hypothalamic-pituitary-adrenal axis rapidly recovers following discontinuation of short-term corticosteroid therapy, distinguishing this pattern from the prolonged suppression associated with chronic corticosteroid use. Other reported adverse effects including gastrointestinal discomfort, dizziness, and headache occur infrequently and are typically mild and self-limiting [9]. Serious adverse events including severe infections, anaphylaxis, or significant metabolic derangements are exceedingly rare in the context of perioperative corticosteroid use.

## Conclusion:

Corticosteroid therapy represents an effective and well-established intervention for reducing postoperative morbidity in oral surgery. Available data shows that significant reductions in pain, facial swelling, and trismus with favorable safety profiles when appropriate dosing regimens are employed. A single preoperative dose of 8 mg dexamethasone administered 30-60 minutes before surgery emerges as an optimal evidence-based protocol, offering robust clinical benefits with minimal risk of adverse effects.
